# Fault Detection and Diagnosis in Multi-Robot Systems: A Survey

**DOI:** 10.3390/s19184019

**Published:** 2019-09-18

**Authors:** Eliahu Khalastchi, Meir Kalech

**Affiliations:** 1 Computer Science School, College of Management Academic Studies, Rishon LeTsiyon 7579806, Israel; 2 Software and Information Systems Engineering, Ben-Gurion University of the Negev, Beersheba 8410501, Israel

**Keywords:** fault detection, fault diagnosis, multi-robot systems, survey

## Abstract

The use of robots has increased significantly in the recent years; rapidly expending to numerous applications. These sophisticated machines are susceptible to different types of faults that might endanger the robot or its surroundings. These faults must be detected and diagnosed in time to allow continual operation. The field of Fault Detection and Diagnosis (FDD) has been studied for many years. This research has given birth to many approaches that are applicable to different types of physical machines. However, the domain of robotics poses unique requirements that challenge traditional FDD approaches. The study of FDD for robotics is relatively new; only few surveys were presented. These surveys have focused on the single robot scenario. To the best of our knowledge, there is no survey that focuses on FDD for Multi-Robot Systems (MRS). In this paper we set out to fill this gap. This paper provides detailed insights to the world of FDD for MRS. We first describe how different attributes of MRS pose different challenges for FDD. With respect to these challenges, we survey different FDD approaches applicable for MRS. We conclude with a description of research opportunities in this field. With these contributions it is the authors’ intention to provide detailed insights to the world of FDD for MRS.

## 1. Introduction

The use of robots in our daily lives is increasing. Recent reports by the International Federation of Robotics [1] describe yearly increases of 15% and 25% in sales of service and industrial robots respectively. The use of robots is appealing for tasks that can be referred as the four D’s—too Dangerous, too Dull, too Dirty and too Difficult—to be done by humans. Such tasks include surveillance and patrolling [2], aerial search [3], rescue [4], and mapping [5]. Robots are physical systems with varying degrees of autonomy that operate in different and dynamic physical environments, e.g., satellites; Martian rovers; unmanned aerial, ground, or underwater vehicles; etc. Like any physical system, these sophisticated and sometimes very expensive machines are susceptible to different types of faults such as wear and tear, noise, or control failures [6]. However, robots differ from other physical systems in purpose, design, and control. These differences expose robotic systems to additional types of faults, and to unique fault impacts. As a result, these differences pose constraints and requirements that challenge traditional Fault Detection and Diagnosis (FDD) approaches. In addition to these challenges, FDD becomes significantly more complex for Multi-Robot Systems (MRS).

The motivation for FDD in physical systems in general and particularly in robots is to facilitate recovery from damage caused by faults. In the robotics domain, faults have the potential to affect the robot’s efficiency, lead to the robot’s failure, and even jeopardize the safety of the robot or its surroundings [7]. For instance, a UAV (Unmanned Aerial Vehicle) can suffer a fault in the case of a stuck aileron (controls the roll axis of an aircraft). The compensating actions of the UAV’s autopilot prevent failure, but the fault may cause more drag on the vehicle which increases fuel consumption, thereby compromising the robot’s efficiency [8]. Faults such as an engine failure, wing-icing that leads to a stall, or faulty altitude control might cause the UAV to take a steep dive—thereby failing to fulfill its mission and endangering the safety of the UAV and its surroundings. In addition, faults have the potential to propagate through the multi-robot system. For instance, the occurrence of a navigation fault to a formation-leading UAV might cause the entire formation to fail its mission. When a fault is detected, it is important to proceed with a diagnosis process to identify which individual robots and perhaps which of their internal components are involved. This diagnostic information can be used for recovery or for decision making purposes such as using undamaged redundant systems or re-planning [9]. The ability to recover successfully allows the system to maintain its reliability, robustness, and efficiency which is ultimately the reason for applying FDD.

The challenge of FDD for MRS includes several aspects. First, global system knowledge depends on local beliefs of individual robots (derived from their sensors). An FDD mechanism is expected to identify knowledge inconsistencies and associate them to individual faulty robots. Second, the process of global decision-making and the creation of a global multi-robot plan may result in a faulty plan; i.e., some steps of the plan are wrong. An FDD mechanism is expected to identify such steps. Third, the robots derive their own local plan in support of the global effort, and then, the robots coordinate their execution. An FDD mechanism is expected to monitor the execution of the MRS plan and diagnose whether local planning, coordination, or local action is the source of faulty execution. Finally, after the identification of an individual robot, a complete FDD mechanism is expected to diagnose the faulty components of the robot. In this paper, we focus on plan related faults and coordination related faults. These faults are at the core of the “multi” aspect of multi-robot systems and encompass a variety of other types of faults.

The attributes of different multi-robot systems challenge FDD in different ways. In this paper we focus on two main attributes. The first attribute is the degree of collaboration. Namely, a higher degree entails greater dependency on global knowledge, planning, and coordination of execution. An FDD mechanism is hence challenged to diagnose in which of these elements a fault might reside. The second attribute is the size of the MRS. Namely, as the number of robots increases thus monitoring becomes more challenging. In particular, we focus on robotic swarms, i.e., a swarm-size of very simple robots. In the domain of robotic swarms, some of the above challenges are taken to the extreme.

The study of FDD for robotics is relatively new. In recent years, a few surveys [10,11] have been presented. These surveys have focused on traditional FDD approaches and how these approaches may apply to a single robot scenario. To the best of our knowledge, there is no survey that centers on FDD for MRS.

In this paper, we strive to fill this gap by surveying different works about plan related faults, coordination related faults, fault tolerant MRS architectures, and the special case of FDD for robotic swarms. The rest of the paper is organized as follows. In Section 2 we set the stage for our discussion, we provide a description of a general MRS and how it differs from a single robotic system and from a system of multi software-agents in the context of FDD. In Section 3 we analyze how different attributes of MRS pose different challenges for FDD. In Section 4 we provide the detailed survey of different FDD approaches applicable for MRS. In Section 5 we describe research opportunities in the field. With these descriptions it is the authors’ intention to provide detailed insights to the challenging world of FDD for MRS.

## 2. A Multi-Robot System in the Context of FDD

In this section we describe a multi-robot system in the context of fault detection and diagnosis requirements. In particular, in Section 2.1, we shortly summarize FDD for a single robot scenario, and in Section 2.2 we describe MRS in the context of FDD. Namely, we describe how MRS differs from the single robot scenario and from a multiagent system of software agents.

### 2.1. FDD for Single Robotic Systems: A Short Summary

As mentioned in the introduction, in this paper we discuss the fundamentals of FDD for multi-robot systems. This work is complementary to our introductory work on the fundamentals of FDD for single robotic systems [12] which we shortly summarize in this section.

**Figure 1:**
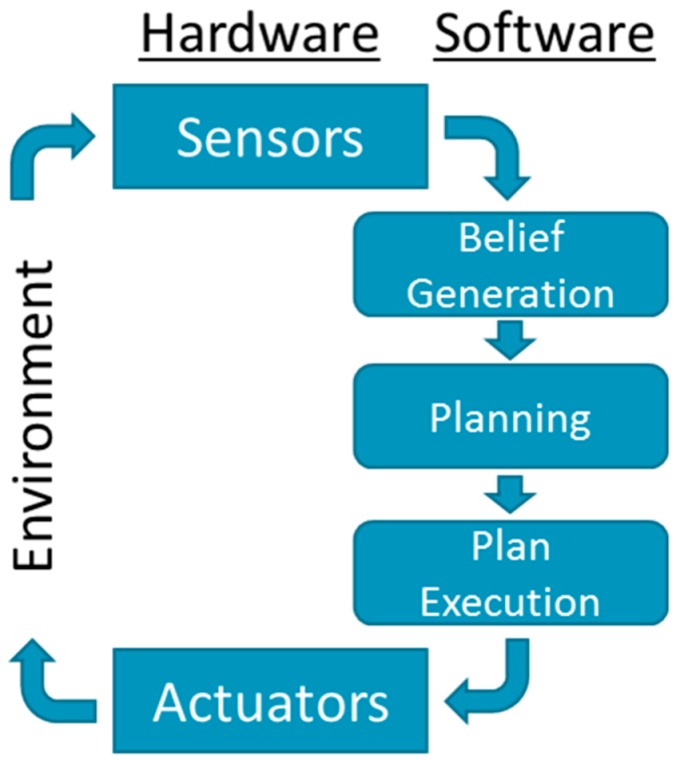
A general control flow of a single robot.

A robotic system (see Figure 1) is an embodied physical agent which operates in the physical environment in order to achieve a certain goal. A robotic system is a system comprised of both physical and virtual components. The physical components (hardware) are made of (a) sensors that allow the robotic system to assess the state of the robot and the state of the local physical environment, (b) actuators that allow the robotic system to manipulate the state of the robot and the state of the local physical environment, and (c) other hardware components such as a power supplier and a computer.

The computer supports the virtual components of the robotic system (software). These components perform the tasks of (a) processing sensory data into useful information (belief generation), (b) using the information to derive an intelligent decision about how to act (planning), and (c) issue the instructions that support the decision (plan execution). These three major tasks are performed in continual loop until the goal is achieved and are known as the *sense-think-act* cycle [13]. For example, the robot (1) uses an image processing technique on the data returned by a camera sensor to recognize the relative location and momentum of a desirable object, (2) inputs this information to a decision tree and decides to approach the object at a certain planned speed and heading, and (3) issues instructions to the wheels such that the robot will approach the object. These three tasks are performed in a continual loop since as the object and the robot move different decisions must be made and applied.

Faults have the potential to occur in every physical or virtual component of the robotic system, effectively propagating through, and disrupt the *sense-think-act* cycle of the robotic system. Thus, faults have a significant negative impact on the ability of the robotic system to fulfill its goal. Moreover, some faults might endanger the system and its surroundings, e.g., a fault to the engine of an Unmanned Aerial Vehicle (UAV) might lead it to crash. Different FDD approaches are used as complementary mechanisms that may allow a recovery after a fault is quickly and accurately detected and diagnosed. However, different FDD approaches encounter different challenges when applied to different robotic systems.

**Figure 2:**
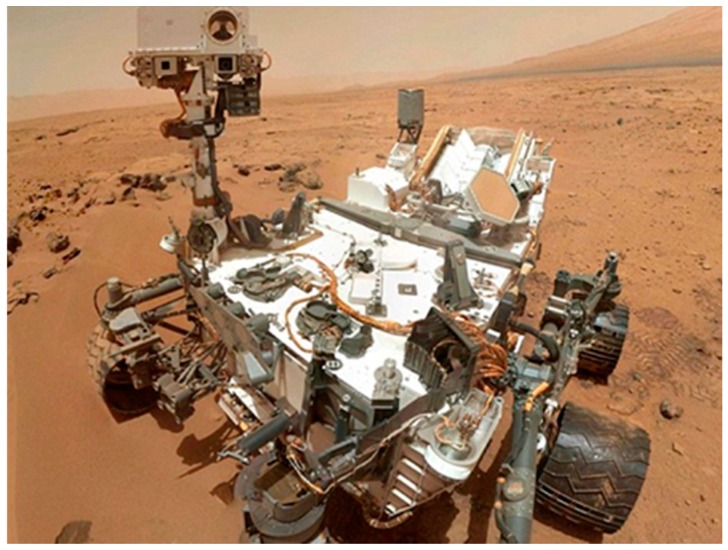
NASA’s Mars Science Laboratory—Curiosity.

To capture the wide range of (single) robotic systems, we introduced four general measures by which the different robotic systems can be characterized. Each measure was depicted as a range where the higher a robotic system is characterized within the range; thus FDD becomes more challenging. The measures we defined are (1) the degree of general use, (2) the degree of autonomy, (3) the degree of complexity, and (4) the degree of the interaction with the environment. For instance, the Mars rover Curiosity (see Figure 2) is highly autonomous since communications to Earth may take up to 22 minutes. The autonomy creates a number of challenges for FDD. The FDD mechanism cannot rely on the concurrent external observation of a human operator; it must rely on the sensory data of the robot to detect faults. These sensors carry a degree of uncertainty and might even be faulty themselves. The FDD task, as other time-critical tasks, must be done by the robot itself. FDD cannot rely on remote support systems; all FDD computations must be done locally. In order not to interrupt other mission-oriented computations, the FDD task must be computationally light weight, while still being quick and accurate. Such requirements challenge different FDD approaches in different ways. Our full analysis of the different measures and their impact on FDD approaches will be available for reading in [12].

### 2.2. A Multi-Robotic System

A multi-robot system consists of multiple robots working as a system, i.e., robots that maintain prolong interactions or interdependencies in order to form a complex whole and achieve a common goal within a physical environment. To better understand the attributes of an MRS we compare MRS to a single robotic system and to a Multi-Agent Systems (MAS) of software agents.

**Figure 3:**
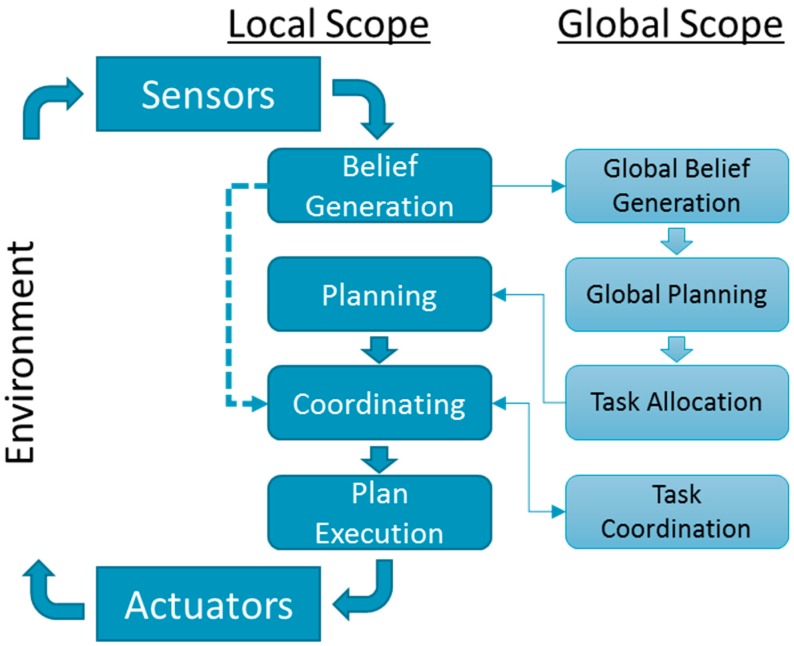
A general control flow of a single robot in an MRS.

In a way, MRS can be considered as a distributed robotic system that senses, thinks, and acts. However, each of these processes becomes significantly more complex in MRS than in a single robotic system. Figure 1 depicts a general control flow of an MRS as a relationship between the local scope of an individual robot and the global scope, which is typically shared with the other robots. Different MRS architectures such as [14,15,16,17], which we survey in Section 3, differ in implementation and focus. In some architectures the global scope is centralized in one robot; in others, it is shared or distributed. Existing MRS Architectures focus on task allocation mechanisms, distributed resource sharing and management, or coordination mechanisms [17]. Nonetheless, all architectures possess, to some extent, the general elements depicted in Figure 3.

Each robot senses its local environment and generates individual beliefs. These beliefs are communicated to the global scope for global belief generation. Individual robots may not know about the local beliefs of others. In addition, individual robots may not communicate all their beliefs. Global belief generation must collect the local beliefs, assess if the MRS has sensed all it needs to, and reason about whether the perceptions of the robots are even truthful [18].

Thinking becomes more complex as the robots must construct and maintain a global model of knowledge based on the local information brought by each robot. The global model of knowledge allows the MRS to make intelligent decisions and perform global planning. Unfortunately, in the unforeseeable dynamic nature of the physical environment, it is infeasible to have the perfect knowledge and computational resources required to create globally accurate models [14]. This is a local challenge to each individual robot and a global challenge to the MRS.

After a global plan is selected, tasks are allocated to individual robots (via communications). Then, each robot applies local planning in support of the global plan by selecting the steps necessary to successfully complete its assigned task. However, before the execution of the local plan, the robot must coordinate its actions with the other robot in the system. That is, how to execute the planned steps under the constraints of time and order with respect to the other robots. After the robots agree and can coordinate, the individual robot executes its local plan.

As the robot operates, it affects the environment which is again being sensed as the sense-think-act cycle depicts, and the process repeats. However, if a new task is not assigned and the local plan is not changed, then the local beliefs are fed straight into the coordination mechanism (dashed line of Figure 3), maintaining coordination as execution continues. Each of these local and global activities might suffer from different faults, which in turn, disrupt the entire MRS.

MRS can be regarded as a private case of a Multi Agent System (MAS). However, it would be wrong to assume that approaches that are dedicated to MAS and apply to software agents can be applied to MRS. In every work concerning MRS, there is a strong emphasis on the differences which make such approaches inapplicable to MRS. Three major differences are typically discussed: (a) the dynamic nature of the physical environment in which the robots (embodied agents) operate brings uncertainty to sensing, environment modeling, and actuating; (b) robots possess limited computational power which is consumed by basic tasks such as vision processing; and (c) there is limited computational time since most operations are time-critical. These differences challenge approaches for MAS which are not dedicated for MRS. In particular, an FDD approach would have to meet an additional challenge: The space of possible faults is significantly larger in MRS than in a software MAS. Nonetheless, there are a few aspects of FDD for MRS that can be met by FDD approaches for MAS, as we survey in Section 4.

MRS are different in their degree of collaboration, coordination, communication, size, redundancy, and other attributes. Each attribute has its own impact on fault susceptibility and on FDD mechanisms, e.g., centralized vs. distributed FDD mechanism. On the other hand, MRS offer social diagnosis, and the distribution of the FDD mechanisms such that it is scalable. We cover these issues in the next sections.

## 3. Attributes of MRS and Their Impact on FDD

In this section we describe how different attributes of MRS challenge FDD mechanisms in different ways as inferred from the survey presented in Section 4. In particular, we discuss in Section 3.1 two general types of faults that MRS might encounter. We continue with different attributes of MRS which impact FDD: (1) the collaboration factor in Section 3.2, (2) the size factor in Section 3.3, and (3) the swarm factor in Section 3.4.

### 3.1. The Typical Faults and Their Impact on MRS

Each of the local and global activities depicted in Figure 1 might suffer from different faults, which in turn disrupt the entire MRS. In [19], Parker includes the following faults an MRS might encounter: individual robot malfunctions, local perspectives that are globally incoherent, inter-robot interference, software errors or incompleteness, and communications failures. In this paper we focus on two fundamental types of faults, which are at the core of the “multi” aspect of multi-robot systems: planning related faults and coordination related faults. The other types of faults described in [19], such as communication failures or incorrect local perceptions, will eventually evolve to or be the result of either planning related faults or coordination related faults.

**Planning related faults** depict the occurrence of faulty plan-steps either in the global or the derived local plan. A faulty plan-step is an instruction which either leads to mission failure, degraded performance, or waste of resources such as energy. For instance, in a foraging domain a faulty step of the global plan resulted in the following task allocation: one robot is idle, and two robots forage in the same area, interfering each other. Even though each robot is healthy, this fault causes degraded performance of the MRS. An FDD mechanism is challenged to identify the faulty plan-step as the root cause. It is challenging since there are many other alternative explanations for the degraded performance. For instance, the idle robot falsely reported it is engaged in another task, or the two foraging robots cannot coordinate due communication failures, etc. Viewing the plan as a system to be diagnosed may uncover faulty plan-steps or even lead to a cause rooted in a particular robot.

**Coordination related faults** depict any fault that might disrupt or prevent the coordinated execution of the robots’ plans, i.e., the execution of a local plan under the constraints of time and order with respect to the rest of the MRS. The robots must agree on their beliefs and execute their local plan in a coordinated fashion. Communication-depended architectures for MRS may find communication failures as the most likely source of coordination faults. However, faults such as incorrect local perception, overloading the robot’s CPU and memory, faulty actuators and many other types of faults, might also cause coordination faults. An FDD mechanism is challenged to distinguish coordination faults from planning related faults and diagnose the root cause of these faults.

The impact of faults in the global plan is significant, task allocation is compromised, and the robots perform undesired tasks that in turn degrade the MRS performance or even lead it to fail. Faults in a local plan lead to incorrect action, which in turn might degrade the performance of the MRS. The impact of coordination faults is significant as well. Without coordination, robots might interfere, delay, or even prevent the actions of other robots in the system. A complete FDD mechanism must diagnose the plan-step, individual robot, or the related component of an individual robot which serves as the source of the degraded performance or failure of the MRS.

Next, we discuss several attributes of MRS and their impact on FDD.

### 3.2. The Collaboration Degree of an MRS and Its Impact on FDD

Different levels of collaboration in a multi-robot scenario introduce different attributes that can either contribute to or challenge fault detection and diagnosis. In a multi-robot scenario robots may (1) collaborate towards a shared goal [2] or (2) cooperate towards selfish or altruistic common goals [20], or anywhere between these two extremes. Regardless of which, the robots may or may not exhibit coordinated behavior.

When **collaborating** towards a shared goal the robots act as a team. The team’s performance is heavily dependent on the team global plan and the ability of each team member to understand the team goal, the team plan, and the roles of other team members. In addition, each team member must derive and execute its own supporting role and even help or cover for other team members if they malfunction.

**Figure 4:**
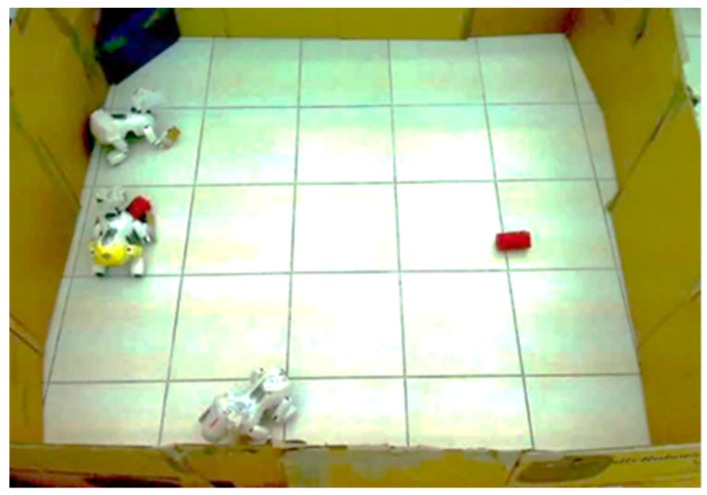
Sony AIBO robots foraging red items.

For example, a team of foraging [21] robots should search and collect resources (see Figure 4). Consider that two robots are headed towards the same resource due to a faulty plan-step or coordination fault. Fortunately, other teammates may recognize this behavior as faulty and communicate this information to these two robots and aid in improving the team’s performance.

On the positive side, team awareness is helpful for social fault detection and diagnosis [22,23,24,25] A team member has an expectation of how the other team members should act or what they are trying to achieve. If a team member acts in an unexpected manner, then other team members may recognize this as a fault. Nevertheless, sharing and executing a team plan introduces a large variety of weak points that a fault diagnosis mechanism should take into account. These weak points include interdependencies, communications, synchronization, new information handling, etc. As the size of the team increases so does the number of weak points that the fault diagnosis should consider.

In a multi-robot scenario, robots may **cooperate** to achieve selfish or altruistic common goals [20,26]. In this scenario a robot is not heavily dependent on the knowledge about the other robots; it is only dependent on the ability to achieve cooperation when needed. This requires a higher form of cognitive ability, usually applied with artificial intelligence methods and game-theory reasoning [27]. A robot may help other robots in exchanging needed resources to achieve its own desired goals. A robot may negotiate [27] with other robots and choose to cooperate with the robots that are most beneficial to the task at hand. Since there is less dependency on a prior global plan, we can expect less global plan related faults, but the local plan might still be faulty. During the cooperation, there is typically heavy reliance on coordination, and hence, we can expect coordination faults.

**Figure 5:**
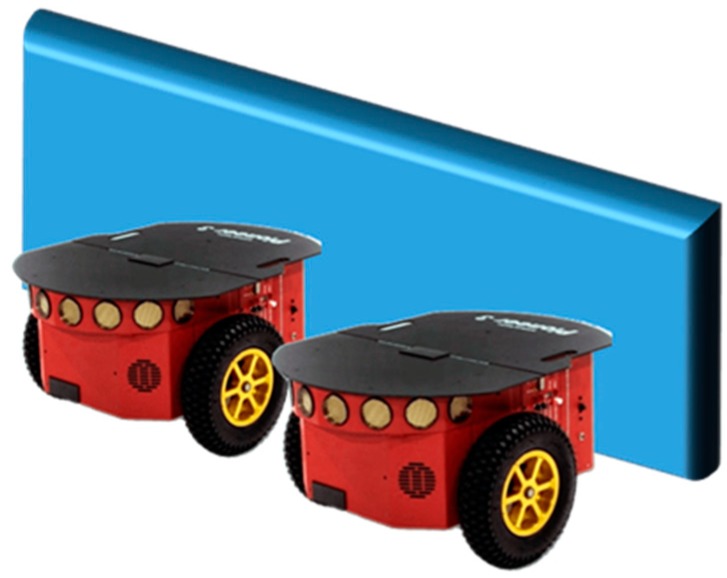
Illustration of two pioneer robots pushing a wide box as in [27].

For example, consider two robots that are supposed to move through a path that is discovered to be blocked by a wide obstacle (see Figure 5). One robot cannot move the obstacle out of the way on its own. The two robots may decide to cooperate and move the obstacle together in order to clear their path and continue with their goals. The robots coordinate together how they should move the obstacle to the left and assign each robot with a different side of the wide obstacle, i.e., left and right. Therefore, the right robot individually derives a local plan to push faster than the left robot, causing the box to move to the left. However, both robots appear to be moving at the same speed and the obstacle is pushed straight along the way and not out of the way to the left.

This observation may be explained by coordination related faults such as: (1) the robots did not agree on the desired speed, (2) the command to start was delayed due to a communication fault, and (3) the left robot did not recognize the right robot and thus was unable to adjust its speed. In addition, a local plan related fault may explain this behavior, e.g., the right robot did not calculate the correct desired speed. Fortunately, one of the robots may detect that the desired goal is not being achieved in the expected time and decide to cooperate with another robot or to re-coordinate with the same robot.

As opposed to a collaborating team of robots, cooperative robots have less prior information about the other robots. The information is acquired during cooperation [20]. The social diagnosis capabilities are limited to the amount of information the robots are willing to share during the cooperation. In addition, the cognitive capability to cooperate with different robots is a complex control feature on its own that further challenges fault isolation in the individual robot.

When the robots act in a coordinated fashion, a pattern may emerge from the harmonious movement of individuals or groups of robots. A fault detection mechanism can exploit such patterns to trigger diagnosis when these patterns suddenly disappear. This is mostly useful when the robots exhibit a formation-maintenance behavior. A formation is a geometrical structure that emerges out of the positions of the robots with respect to each other, which is maintained even during movement. As such, formations require high degrees of coordination. The formation generates an expectation that planning or coordination related faults may easily break. As the desired formation is not kept, diagnosis can be triggered [28].

**Figure 6:**
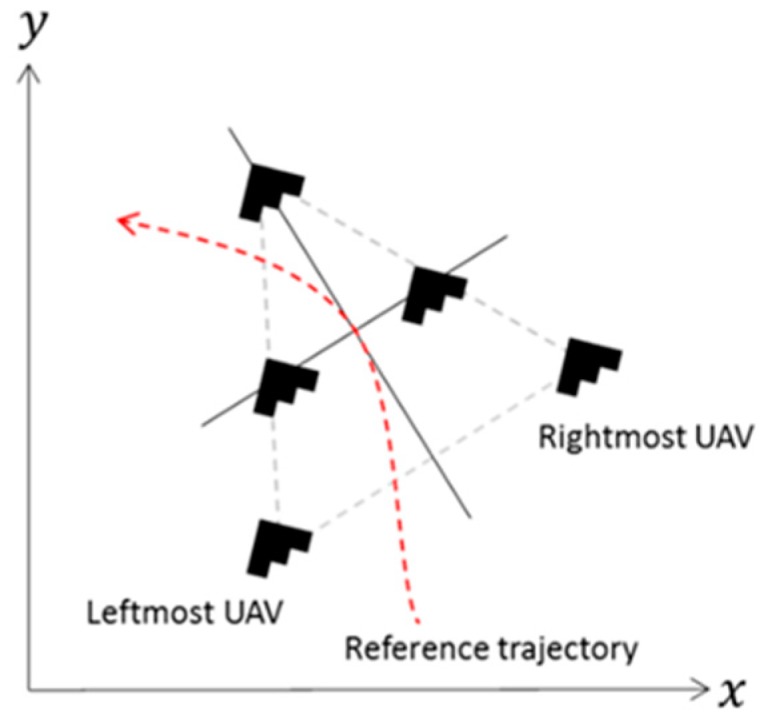
UAV formation taking a left turn.

For example, consider several UAVs that are coordinated to fly in a tight formation (see Figure 6). The formation element acts as one piece that has characteristics such as altitude, speed, and heading. Individual UAVs in the formation may have different, yet similar, characteristics of altitude, speed, and heading for keeping the tight formation element together, e.g., while taking a left turn the rightmost UAV should fly faster and higher than the leftmost UAV. Consider an observation that the formation breaks. Since the formation is expected to be kept, this observation may indicate a fault.

In this example, the first challenge is to decide when the formation is considered as broken. This aerial formation is dynamic in nature, and slight position deviations may occur. Individual UAVs are keeping the formation by maintaining control of these deviations. A fault that is reported upon detection of these small deviations is most likely to be a false positive. On the other hand, large deviations may be the result of an expected structural change of the formation, e.g., shifting from an aero head shape to a column shape. A fault that is reported upon detection of these large deviations is most likely to be a false positive as well.

The second challenge is to decide whether the fault resides in an individual UAV or in their coordination. Consider an observation that the rightmost UAV drifts out of the formation while the formation is taking a left turn. The fault may reside in the rightmost UAV, e.g., faulty controls, faulty sensors, or even faulty understanding of its role in the coordination, for instance, which UAV to follow. Alternatively, the fault may be caused by a coordination related fault. For instance, the command from the formation leader was delayed to the rightmost UAV, or the rightmost UAV has lost its leader.

### 3.3. The Size of an MRS and Its Impact on FDD

As discussed previously, a shared team plan exposes additional weak points that an FDD mechanism should consider, burdening fault isolation. (1) When the team plan is more complex, the fault isolation process becomes even more challenging as there are more elements to consider. (2) Fault detection becomes more challenging due to the mere fact that there are more individual robots to monitor. Moreover, (3) more robots produce more data that may yield information which may contribute to an FDD mechanism. However, considering the large amount of data that the robots provide, it is very challenging and sometimes impractical. An FDD mechanism is challenged to dismiss data which may be irrelevant and extract the quality information that is useful for fault detection and diagnosis. The challenge is to maintain scalability as the data sources are increased; this is especially true for data driven approaches. One of the ways scalability is achieved is by distributed FDD algorithms as we discuss in Section 4.

Having more robots yields greater redundancy. Social diagnosis becomes an available opportunity. The FDD task can be decentralized to be done by several robots making the FDD task more scalable [29]. In addition, different robots may apply different FDD approaches making the FDD task more robust. Finally, distributing the diagnosis task among multiple robots prevents a single point of failure by letting the non-faulty robots to complete the tasks of the team that might have failed contributing to fault tolerance and recovery.

### 3.4. The Impact of a Robotic Swarm on FDD

A special case of a large size of robots in a multi-robot scenario is a **swarm robotic system**. Research on swarm systems has attracted much attention in recent years [30]. A robotic swarm system is a system that consists of multiple intelligent interconnected robots and possesses swarm capabilities. A swarm system consists of the robots and the topology of the robots. The main characteristics of a robotic swarm system are: (1) no centralized control, (2) low intelligence of individual robots, (3) local interconnection among robots, and (4) the emergence of swarm intelligence out of their basic behaviors.

**Figure 7:**
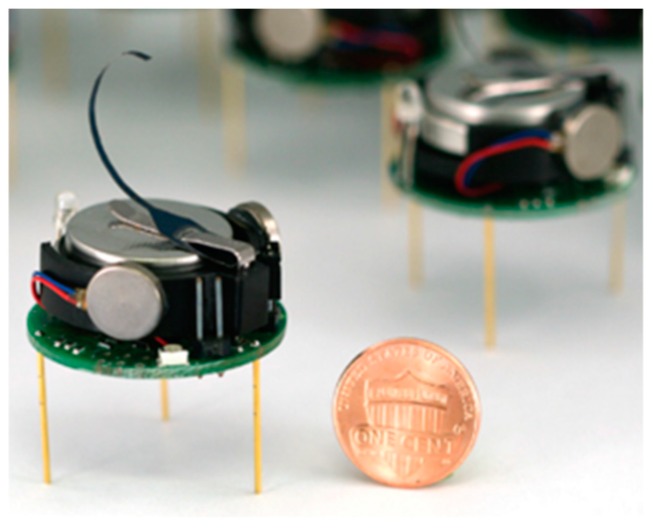
An individual Kilobot [31].

**Figure 8:**
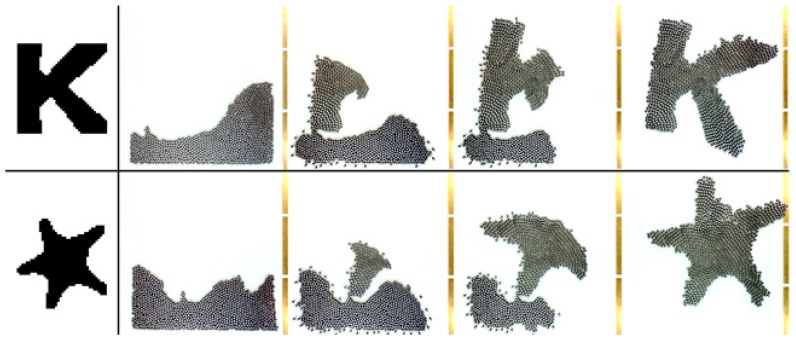
A swarm of 1024 Kilobots gradually arrange themselves into a ‘K’ shape and a star shape [31].

(1) The swarm behavior emerges out of the local behavior and interactions of each individual; there is no centralized control to guide their behavior. (2) The intelligence of individual robots is relatively low. For instance, the tiny swarm-individuals depicted in Figure 7 cannot process complex computations. The robots can process local data by themselves and may disregard any global consideration. Their individual behavior is affected only by their local context, which includes local sensing and the behavior of the robot’s neighbors. The swarm behavior emerges from simple individual behaviors which are relatively easy to model [31]. (3) The interconnection among robots means that the robots are able to exchange messages only with other neighboring robots in a swarm system. (4) The swarm intelligence emerges from the coordinated behavior of local robots in the swarm. For example, Figure 8 depicts how 1024 simple Kilobots apply basic behaviors to slowly form a ‘K’ shape and a star shape [31].

Consider the following more intricate example. Assume a maze should be mapped. Assigning this task to a multi-robot team would force them to create a team plan and assign a different role for each team member. The team members would have to communicate as they cover the maze, reassigning roles as they go. If one robot malfunctions, the other teammates would have to re-plan and take over its assigned role. On the other hand, a swarm of robots can enter the maze while executing a very simple behavior dispersal. This behavior causes the robots to scatter while each is trying to stay as far as it can from its sensed neighbors. The sheer number of robots in the swarm makes them cover the entire maze-like water or gas particles. It would take a critical mass of malfunction robots to interfere with the maze cover mission.

Early research considered swarm robotics to be fault tolerant due to the inherited redundancy [32]. Recent research [33] shows that failed robots can and will significantly affect healthy robots and lead to task failure. Since an individual robot reacts to the observed behavior of its neighbors, fault symptoms are transferred among neighboring robots and cascade along the swarm. Faults in swarm systems can be classified into two types according to their influences: (1) *Topology Faults*—change the swarm topology, and (2) *Component Faults*—affect only actuators, sensors, or controllers of the robot [30].

For example, assume a robotic swarm is tasked with a search and rescue mission. First, the swarm is ordered to disperse in order to cover as much space as the swarm can. If an individual robot finds the survivor, it lights an infrared beacon. Neighboring robots transmit the information that they can see the beacon in a neighboring distance of 1. Any other robot that receives transmitted messages with values $V=\left\{v_{1},\ldots ,v_{n}\right\}$ will transmit the value $\underset{v_{i}}{\min}V+1$. Thus, a relative short path to the survivor can be extracted from always choosing the path to the neighbor that has the closest neighboring distance to the survivor. Consider one robot that cannot move. This is a component fault that will not affect the topology of the swarm very much and is less likely to damage the swarm performance; the swarm can tolerate such faults. On the other hand, consider one robot that accidently lights its beacon. This topology fault will lead the swarm to a false position of the survivor.

Even though each robot in the swarm is quite simple, fault detection and diagnosis are not. The challenge that FDD mechanisms face is to isolate the robot that is seeding a topological fault that might lead to the failure of the swarm. The seeding of a topological fault can start with very tiny deviations in an individual’s behavior that might be undetectable. On the one hand, monitoring every individual robot and generating an expected behavior for each one is a very hard task since (a) there are too many robots to monitor, and (b) the context in which the robots operate may be highly dynamic. The context of an individual robot in a swarm is its local neighbors and their actions. The frequency at which an individual robot makes decisions and changes its behavior and state may be very high. In order to predict the robot’s behavior, the data sampling rate should match this very high frequency. Otherwise, in between two data samples, the robot may have already changed its state several times, making it impractical to predict its behavior. Sampling large amounts of data at very high frequency poses processing challenges for an FDD mechanism.

On the other hand, monitoring the swarm as one body is relatively easier, and it allows the detection of faulty swarm behavior. For instance, features like the swarm boundaries, shape, speed, and heading can be extracted and be exploited for fault detection, e.g., instead of bypassing an obstacle, the robots have formed a ring around it and continue to circle the obstacle. However, the isolation of the seeding robot (or robots) among the entire swarm becomes more challenging when viewing the swarm as one body, e.g., every robot does execute its follow instruction correctly, and the robots which have closed the circle around the obstacle cannot be recognized anymore.

Finally, since the swarm is comprised of simple robots with no centralized control, the implementation of the FDD approach becomes an issue. FDD should be distributed, and yet each individual carries limited computational power and is exposed only to local information. The main changes in implementing an FDD mechanism for robotic swarms are: (1) how to gather and share global information (2) in a scalable distributed manner and (3) apply the FDD algorithm on the computationally weak robots.

### 3.5. Summary of the MRS Attributes and Their Impact on FDD

To conclude, An FDD mechanism in a multi-robot scenario is challenged to detect and diagnose plan related faults and coordination faults. As the level of collaboration increases, there is a higher capability for social fault detection and diagnosis, yet there are additional weak points in the team and the individual plans and their coordinated execution that the FDD mechanism should consider. Formation-maintenance behavior generates an expectation that is useful for fault detection. As the number of participating robots increases, thus it becomes more challenging for an FDD mechanism to monitor the robots, process their data, and isolate faults in their complex team plan. However, the following advantages are gained: (1) social diagnosis is enabled, (2) the FDD task can be decentralized and become more scalable, and (3) different FDD approaches can be applied, making the FDD more robust. In a robotic swarm system, an individual robot is not complex; it is built of simple means and displays simple behaviors. Nonetheless, the challenge is to detect and diagnose the robot that is seeding a topological fault that might lead to faulty swarming behavior.

## 4. A Survey of FDD for MRS

The works we survey here are categorized according to the attributes of MRS presented in Section 3 and their impact on FDD. In particular, in Section 4.1, we discuss works which handle planning related faults. We continue with works which handle coordination related faults in Section 4.2. Fault tolerant architectures are discussed in Section 4.3. We conclude with FDD for swarms which is discussed in Section 4.4.

### 4.1. Diagnosis of Plan Related Faults

Here we cover works which are related to the diagnosis problem in multi-robot systems plan; i.e., the plan performed by the robots is considered as a system to be diagnosed. Under this context, a multiagent system and MRS can typically be interchangeable. Roos and Witteveen [34] investigated this problem. They introduced a formal model where partial observations of plan states are compared with predicted states based on normal plan execution. Deviations between observed and predicted states can be explained by faulty planning. The diagnosis is a subset of abnormal plan steps that can explain the incompatibility between the predicted and the observed state. They show how these diagnoses can be found efficiently if the plan is distributed over a number of agents.

Extending this notion, de Jonge et al. [35] introduce the use of model-based diagnosis in two general types of plan diagnosis: primary plan diagnosis identifies the incorrect or failed execution of actions, and secondary plan diagnosis identifies the root cause of these faulty actions. The primary diagnosis is linked to the secondary diagnosis and thus the root cause (e.g., agent, equipment) of failed plan steps can be diagnosed.

Micalizio [36] proposed a distributed approach to autonomous plan repair. Each agent executes a local plan that is derived from the global multiagent plan. The agents autonomously monitor, diagnose, and repair their local plan. Since the system is only partially observable, the state of an agent is not certain but rather estimated as a set of possible states. Thus, the diagnosis is typically ambiguous, and thus the repair re-planning step must handle uncertainty. They show that the proposed methodology is adequate to promptly react to an action failure and that the computational cost of the approach is affordable since the agent diagnosis highly constrains the search for a recovery plan.

In later work, Micalizio and Torasso [37] presented a novel methodology named Cooperative Weak-Committed Monitoring (CWCM) where the diagnosis of the multiagent plan, executed in a dynamic and partially observable environment, is addressed in a fully distributed and asynchronous way. As opposed to previous approaches the action failures are not assumed as independent of each other. CWCM exploits nondeterministic action models to carry out two main tasks: detecting action failures and reconstructing possible beliefs an agent has had about the environment. Thus, each agent has the ability for self-diagnosis in terms of explaining action failures as exogenous events. A diagnostic engine is utilized for distinguishing primary and secondary action failures. They show that CWCM is effective in identifying and explaining action failures even where the observability of the system is significantly reduced.

Generally, when diagnosing the multiagent plan, the recovery may reside in re-planning. As stated in our introduction, re-planning is a sensitive subject in MRS; one must consider the possibility that by the time re-planning was made the dynamic environment had already changed, forcing the need to re-plan again. A potential solution can be found in the work of Stancliff et al. [38]. They argue that the MRS plan must consider in advance robot failures instead of re-planning after the fact. This consideration of robot failures includes backup plans for task allocation; when tasks of failed robots are given to healthy robots, it is important not to overload the healthy robots and cause them to fail as well. Stancliff et al. show that indeed such an approach results in substantially better mission performance with respect to re-planning.

### 4.2. Diagnosis of Coordination Faults

Here we cover works which are related to the diagnosis of coordination faults, i.e., faults which prevent or disturb the ability of robots in the system to coordinate their actions.

Early works in this subject depicted centralized architectures. For instance, Micalizio et al. [39] have utilized causal models of failures and diagnoses to centrally detect and respond to single-robot failures and to multi-robot coordination failures. Unfortunately, a centralized architecture can be computationally expensive in terms of communications and run-time, and there is a single point of failure as the diagnosing agent might fail. Roos et al. [40] presented model-based diagnosis methods for spatially distributed agents, where each agent is responsible for diagnosing a different subsystem of the MAS. Every agent makes a local diagnosis to its own sub-system and then all agents compute a global diagnosis. However, while building the global diagnosis set there is an assumption that there are no conflicts between the knowledge of the different agents, i.e., that no coordination faults occur.

The following works of Kalech and Kaminka explicitly tackle the problem of diagnosing coordination faults. Continuing with their centralized approach [41], Kalech et al. [29] introduced a distributed, model-based coordination-failure diagnosis approach. In their work, the coordination between the robots is modeled as a constraint graph. For the diagnosis, they utilized different distributed CSP algorithms (Constraint Satisfaction Problem). They concluded that there is a trade-off between the effectiveness of the algorithms, in terms of communication and computation, and the correctness of the diagnosis that the algorithms produce.

The following year, Kalech and Kaminka [24] introduced a novel design space of coordination-diagnosis algorithms. Their underlying assumption was that different faults might lead robots to disagree. They used the term “social diagnosis” to describe the process that diagnoses the reason why robots disagree. This process is divided into the task of selecting the diagnosing robot and the task of computing the diagnosis. Different methods were implemented for each task, and thousands of diagnosis cases were tested. They concluded that (a) centralizing the diagnosis calculation task is critical in reducing communications and that (b) techniques where robots do not explicitly reason about the beliefs of their peers are preferable in terms of computational runtime.

In [25], Kalech and Kaminka have extended their work to scale well with a high number of robots. The social diagnosis scalability was achieved in two ways: (a) they used communications early in the hypotheses generation process to stave off unneeded reasoning, which ultimately leads to unneeded communication, and (b) by diagnosing only a limited number of representative agents (instead of all the agents).

The following cited works depict approaches which, in our view, may handle coordination faults even though this is not explicitly presented in their paper.

Casual models may be very useful for diagnosing coordination faults. However, one must input such faults into the model. In the domain of robotics, one cannot account for every possible fault; the model needs to adapt as experience is gained. For example, Parker and Kannan [42] presented an adaptive causal model method (adaptive CMM) for fault diagnosis and recovery in complex multi-robot teams. A case-based learning approach is utilized to enable the robots to update their causal model. The causal model depicts associations between high-level behaviors, probable faults a behavior might encounter, and the diagnoses of such faults. A lost following-robot is depicted in their experimental setup; it is an evidence that coordination faults can be diagnosed.

Data driven approaches are model free and have the potential to detect coordination faults. For example, Li and Parker [43] introduced a sensor analysis-based fault detection approach that is used to monitor tightly-coupled multi-robot team tasks. They treat the monitored MRS as a dingle black box with only sensor data available. Clustering techniques are used to produce a probabilistic state transition diagram that depicts the normal operation of the MRS. During execution, the state of the MRS is confronted with the leaned model, and deviations raise fault detection alarms. Their empirical analysis included robots cooperating to push a box, where different communication faults were injected. They conclude that by monitoring the robot team as a monolithic robot, more faults can be detected with respect to the monitoring of each teammate separately. The following year, they introduced an improved approach [44] by using of Principle Component Analysis (PCA).

The combination of casual information and temporal (state transition) information can be handled by bond graphs. As such, bond graphs can be very useful for diagnosis in general, and for multi-robot coordination faults in particular. For example, Daigle et al. [45] presented a distributed approach for qualitative fault diagnosis of coupled mobile robots. They utilized a bond graph to model the scenario of the box pushing robots. They injected different actuator and sensor faults which disrupted the robot’s coordination. They have demonstrated the validity and usefulness of their approach.

### 4.3. Fault Tolerance in Architecture of MRS

In past years, several multi-robot architectures were developed with inherent fault tolerance capability. ALLIANCE [14] is a behavior based architecture designed to enable the team of robots to respond to individual robot failures or failures in communication that may occur at any time during a mission. Each robot grows impatient as time progresses, and tasks remain incomplete, possibly due to some faults. This impatience motivates a robot to replace a malfunctioning teammate. GRATE [15] is an architecture for teamwork based on joint intentions. It includes a cooperation layer which is able to funnel important information, such as the inability to perform a task, to relevant teammates, and thus fault tolerance is gained. STEAM [16] is an architecture which facilitates the monitoring of team performance. The representation of reactive team plans includes specifications of monitoring conditions to determine achievement, achievement inability, or irrelevancy of these plans. When achievement inability is detected, a repair plan is activated.

Dias et al. [46] introduced the TraderBots market-based approach for multi-robot coordination. Each robot is modeled as a self-interested agent and the team of robots as an economy. The robots aim to increase their individual profit by trading tasks. In the process, the robots satisfy team objectives while minimizing costs. In [47] Dias et al. analyzed how the TraderBots approach is robust to three types of failures MRS might encounter: communication failures, partial robot failures, and robot death. The basis for the fault tolerance lies in costs being raised when a task cannot be fulfilled, driving the robots to trade with other functioning robots.

Elkady and Sobh [48] surveyed 20 different robotics middleware. Only two are considered to have fault tolerance capabilities; both are focused on a single robot scenario. Multi-robot support, as well as built-in monitoring capabilities, is possessed by ROS—the Robotic Operating System [49]. Gaining great popularity in recent years, ROS is an open source set of software libraries and tools for building single and multi-robot applications that is widely used in robotics research. ROS is comprised of different processes that act like operating system services. These processes provide hardware abstraction, control of low-level devices, common functionality, and message passing. The processing takes place in processes called “nodes”, which can be visually represented as nodes in a graph. These nodes may interact by passing messages about sensor, actuator, control, state, and planning information. ROS provides a simple fault diagnostic system that can be extended. This diagnostic system is mainly designed for monitoring hardware modules and code execution. One example is the work of Safdar et al. [50] that extended these fault detection and diagnosis capabilities to incorporate both software and hardware FDD.

Kirchner et al. [51] introduced RoSHA—a ROS-based self-healing architecture for multi-robot systems. They define the requirements from a self-healing mechanism for MRS and emphasize the need for resource-efficiency and high degree of configurability. RoSHA is designed to address these requirements. ROS monitoring node is utilized to deliver the status of the other ROS nodes to the monitoring component of RoSHA. This information is analyzed in a simple generic diagnostic component, which can be extended with domain specific plugins. Plan selection and team recovery coordination is achieved with the multi-agent coordination language ALICA [52].

Morais et al. [53] introduced a highly abstract cooperative fault diagnostic method for a team of mobile robots based on ROS at the middle layer and the Jason multi-agent framework [54] at the top layer. The top layer enables the high-level programming of diagnosis requests. For instance, a robot has detected that the odometer reports no progress. It cannot know if the odometer is faulty or if it did not move at all. Another robot can provide its observations and help the faulty robot to diagnose the root cause.

### 4.4. FDD for Swarms

Recently, Winfield and Nembrini [33] have shown that the redundancy of the robots in a swarm cannot be tolerant to all faults; explicit FDD is required. However, relative to MRS, there is little research on explicit FDD for robotic swarms [32]. To date, most FDD approaches for swarms take inspiration from other domains. We survey here several works which depict this notion.

Qin et al. [30] provide a comprehensive survey for fault diagnosis in swarm systems. Their survey is not restricted for robotic-swarms; it applies to other areas of swarms as well. However, their review and discussions are very relevant to FDD in robotic swarms. They classified the possible faults into topology faults and component faults and reviewed different FDD approaches that can be taken under consideration for FDD in robotic swarms.

Christensen et al. [55] presented a decentralized approach for detecting faulty robots in a swarm. Inspired by some species of fireflies, their approach relies on a flashing light system, where each individual synchronizes its flashes according to the flashes of its neighboring robots. In turn, the whole swarm is synchronized, except faulty robots which can be easily recognized. They show that moving robots sync faster than static robots, and that the speed of synchronization is inversely proportional to the swarm density.

Lau et al. [56] focus on specific faults in individual robots that can affect the global performance of the robotic swarm. They presented a distributed approach where each individual robot compares its behavioral data to the data of its local neighborhood in order to self-detect errors. This social comparison was tested with several statistical techniques, such as Z-test, and a density-based bio-inspired algorithm. They show that their approach is practical in detecting different failure modes under dynamic environments.

In his Ph.D. work, Hui Keng Lau [32] investigates different approaches for error detection in robotic swarms. He discusses the potential use of bio-inspired Artificial Immune Systems (AIS) for anomaly detection in robotic swarms, emphasizing their adaptability to dynamic environments as a main advantage over other approaches. In particular, he shows how the e Receptor Density Algorithm (RDA) [57] can be applied to error detection in robotic swarms.

## 5. Research Opportunities for FDD in MRS

**FDD for an ad hoc team of robots:** Stone et al. [20] have challenged the AI community to develop theory and to implement prototypes of ad hoc team agents, i.e., teams of previously unfamiliar agents which bend together and achieve collaboration without pre-coordination. This setting appeals to real-world scenarios such as when unfamiliar agents suddenly must collaborate in order to handle an emergency, e.g., put out a fire. Even though the agents do not know each other, they are expected to assume different roles and collaborate. This challenge has given birth to a great variety of approaches that utilize different methods and focus on different aspects of the challenge. To the best of our knowledge, there has yet to be an approach that utilizes FDD techniques.

FDD is very relevant for this ad hoc team setting. Robots need to assume roles if they believe that they are the best ones for these roles. An ad hoc FDD mechanism can help a robot to decide if its teammate is disrupting the operation of the MRS and if so, replace the teammate and assume the role. Here lies a research opportunity—to develop an FDD mechanism for an ad hoc MRS.

An FDD mechanism for an ad hoc MRS faces several challenges. As we have surveyed, current research for FDD in MRS is focused on plan related faults, coordination related faults, or fault tolerant architectures. The different approaches which handle these different aspects are depended on preexisting knowledge, e.g., the global plan, partial observation of teammates’ beliefs, shared coordination constraints, shared architecture, etc. In the ad hoc setting, team plans or strategies cannot be developed a priori, the robots may be heterogeneous, and communication protocols might be unknown. Thus, new FDD techniques should be developed to address this interesting problem.

**FDD for MRS development time:** As most FDD approaches and control architectures for MRS focus on fault diagnosis or tolerance during operation time, a new research opportunity lies in the use of FDD techniques to reduce the development time of MRS. Under development, especially during testing, many types of faults can occur to an MRS, e.g., incomplete control code or software bugs, hardware wear, communication faults, individual-robot faults, etc. The occurrence of such faults slows down the progress of development as developers typically manually diagnose such faults. A development-time FDD mechanism for MRS should be embedded in the development environments of MRS. There are several works that compare different development environments for MRS, e.g., [48,58]. These works show that some attention was given to fault tolerance during development. This attention is typically in the form of open source philosophy, which allows debugging, and layered application, which allows to focus on mission-oriented code. To the best of our knowledge, there has yet to be an attempt to investigate the correlation between using an active FDD mechanism for MRS and the reduction of development time.

**FDD for improved performance of an MRS:** A team of robots may display degraded performance due to a number of reasons. Private misbeliefs of individual robots, unfitting role assignment, misuse of resources, etc. can all contribute to degraded performance. In a team of robots (or agents), one robot may assume the role of monitoring the team and give correcting instructions aimed at improving the team’s performance. For example, one robot may assume the role of a coach [59]; a coaching robot’s role is to improve the team via communications. It is not a centralized coordinator that instructs the agents exactly what to do at each point of time. Rather, it suggests the robots on how to improve by giving them useful and limited information, general instructions, or altering the team plan. Works about a coaching robot [60] in the Robocup competition typically adjust the strategy of the team or find weaknesses about the opponent team such that they could be exploited.

A new research opportunity lies in the use of FDD techniques to isolate behaviors or beliefs which might degrade the performance of the team. In particular, Model-Based Diagnosis may have the potential to recognize private misbeliefs of individual robots about their own role and about the roles of their teammates. The coaching robot may provide the necessary information to correct these beliefs and in turn, improve the team’s performance.

**FDD for an MRS comprised of a team and a swarm:** Typically in the field, MRS is either a team of intricate robots or a swarm of simple robots. Theoretically, an MRS can be comprised of both, exploiting the advantages of each and solve problems differently. For example, consider the search and rescue problem we discussed in Section 3.4. The swarm can be led by a team of robots to the entry of the maze and then be ordered to disperse inside the maze until the survivor is found. Then, the shortest path is calculated and swarm-robots within this path switch on a light that guides the team of robots to the survivor.

A new research opportunity lies in the creation of such a heterogynous MRS and, in particular, in the creation of an FDD mechanism for it. We have discussed the challenges for each type of MRS. The challenges faced by an FDD mechanism for a mixed team-swarm MRS may be great. The team and the swarm affect each other greatly. When a fault occurs, it might propagate through the entire MRS, and fault expressions may be displayed by the swarm and by the team. Isolating the root-cause can be quite challenging. However, some of the implementation challenges faced by FDD mechanisms for robotic swarms can be met by assigning the FDD role to the more intricate robotic team.

## 6. Conclusions

In this paper we have introduced the challenging world of FDD for MRS. We described MRS under the context of FDD. In particular, we described two general types of faults and their impact on MRS. These types are at the core of the “multi” aspect of MRS, and they incorporate the other types of faults: planning related faults and coordination related faults. With respect to these two types of faults, we elaborated on two attributes of MRS and how they challenge FDD mechanisms—the degree of collaboration and the size of the MRS. In addition, we elaborated on robotic swarms and their special characteristics under the context of FDD.

In Section 3 we provided a detailed survey on FDD approaches for MRS. We covered approaches which deal with planning related faults and coordination related faults. We discussed different fault tolerant architectures for MRS and concluded with a brief survey about FDD for robotic swarms. With these contributions, we filled the gap of a required survey and provided detailed insights to anyone who wishes to engage with this challenging subject.
